# Gutta Percha as a Filling Material

**Published:** 1904-05

**Authors:** W. L. Smituh

**Affiliations:** Macon, Ga.


### GUTTA PERCHA AS A FILLING MATERIAL.

By DR. W. L. SMITUH, MACON, GA.

After consulting a number of authorities on the subject of gutta percha, it seems that the oldest writers have given so little space to this subject in their writings as to have passed it by as almost unworthy of notice. I think this very useful and necessary material should be more carefully looked after, and given a more conspicuous place among the various number of filling materials than it now occupies.

I believe why so few are using it to-day is a lack of a proper knowledge of its manipulative qualities and many failures resulting therefrom. They have grown discouraged and laid it aside as useless. I must say that my first efforts in using it were of a discouraging nature. It is an easy material when once we learn how, when and where to use it, difficult until this familiarity is acquired.

In the preparaton of cavities to be filled with this material, it is necessary to prepare them as thoroughly, as for any other stopping that we desire to obtain good results from. In speaking of the kind that I have observed the most favorable results from, is that which comes to us in sheets, and such as many dentists use in a base plate in mechanical dentistry, pink gutta percha. In selecting suitable cavities for this material, I would use it in the proximal cavities of the cuspids, and in that type that are tinted blue, lateral incisors too frail to fill with gold and in the teeth of children, and in exceedingly sensitive teeth, when I wish to avoid the thermal changes that so often result in the destruction of the pulps, when filling teeth without gold and amalgam, in the buccal cavities of the molars, and especially have I seen splendid results in filling the lower front teeth of children. I would be careful in the preparation of the cavities and protect them with the rubber dam properly adjusted, in all cases when practicable, and liberal separation made to give you plenty of room for your work. With these preliminary steps taken, it is necessary to have a convenient vessel by which you can soften your material by moist heat, and I would like to place special stress on this manner of softening your material, for in this one feature of manipulation depends largely your success. One of the most convenient little arrangements I have observed for this purpose, is a little covered metal cup in the shape of half an orange with a cover soldered to the flat side of the orange shaped cup. At one side is a hole sufficiently large to admit the point of a syringe which is filled with water and injected into this little cup. This is mounted on a small frame and set over the flame of your alcohol lamp, such as you use for annealing purposes. Having previously prepared your gutta percha by cutting into small squares or any convenient form, it is now placed on the little heater and the flame turned up, and is gradually heated until the water begins to boil, which will be observed by the escape of steam in the little opening at the side of the cup; lower the flame a little to prevent over-heating your filling materials, but sufficiently high to keep it soft enough for easy and rapid working. I usually begin these fillings in properly prepared cavities just as though I were using pellets or gold cylinders packing one upon the other with suitable instruments until the cavity is filled flush with the margins, then with a flat burnisher pretty well heated, moistened with a slight coating of glycerine press the material as perfectly in place as it is possible, and wait a few minutes until the gutta percha is hardened sufficiently to finish.

I find no use for special instrueumts in working this material, only such as are found in the outfit of any dentist. Right and left approximal gold pluggers, flat burnishers, and after you have filled your cavity flush with margins allow it to harden long enough to return to its natural temperature, and then with a sharp chisel and sand paper strips proceed to finish as with a gold filling. While you might find the work a little less tedious by using chloroform to smooth the margins, yet it is considered detrimental to a good gutta percha filling, and leaves it porous or spongy; on the other hand the very best possible results are obtained.

